# Single-Center Analysis of Soluble TREM2 as a Biomarker in Coronary Microvascular Dysfunction: A Cross-Sectional Study

**DOI:** 10.3390/jcm14061816

**Published:** 2025-03-07

**Authors:** Yingying Xie, Zhaoxue Sheng, Haoming He, Yike Li, Qiang Chen, Yanxiang Gao, Jingang Zheng

**Affiliations:** 1 Department of Cardiology, China-Japan Friendship Hospital, 2 Yinghua Dongjie, Beijing 100029, China; 2 China-Japan Friendship Hospital (Institute of Clinical Medical Sciences), Chinese Academy of Medical Sciences, Peking Union Medical College, Beijing 100029, China

**Keywords:** sTREM2, coronary microcirculation dysfunction, CMD, CFR, IMR, MRR, RRR

## Abstract

**Background:** The soluble triggering receptor expressed on myeloid cells 2 (sTREM2) is linked to the progression of cardiovascular conditions, but its role in coronary microcirculation dysfunction (CMD) is not yet clear. **Methods:** A cross-sectional observational study from July 2023 to May 2024 was conducted in the China–Japan Friendship Hospital, after registration in the ClinicalTrials database (Registry Name: Coronary Microvascular Dysfunction in Angina Patients With Non-obstructive Coronary Artery Disease (ANOCA-CMD); Registry Number: NCT06503640; Registry Date: 23 September 2022). This cross-sectional study involved 76 subjects, including 55 patients with CMD and 21 without CMD, admitted to the China–Japan Friendship Hospital. CMD was defined by a coronary flow reserve (CFR) < 2.5 or index of microvascular resistance (IMR) ≥ 25. sTREM2 levels were measured using an enzyme-linked immunosorbent assay. Linear correlation analysis assessed the relationship between sTREM2 levels and CFR, IMR, microvascular resistance reserve (MRR), and the resistive reserve ratio (RRR). Univariate and multivariate regression analyses further examined the association between sTREM2 and CMD. Additionally, receiver operating characteristic (ROC) analysis was used to evaluate the diagnostic accuracy of plasma sTREM2 for identifying CMD patients. **Results:** Elevated sTREM2 levels were found in the CMD group. Correlation analysis showed a significant positive relationship with IMR and an inverse correlation with CFR, MRR, and RRR. After adjusting for confounders, sTREM2 was found to be an independent risk factor for CMD [OR = 1.003, 95% CI 1.001–1.007, *p* = 0.008]. ROC analysis revealed a sensitivity of 59.46%, specificity of 90.48%, and an AUC of 0.7677 (95% CI: 0.6481–0.8872, *p* = 0.008) for CMD diagnosis at a threshold of 595.5 pg/mL, indicating good diagnostic performance. **Conclusions:** Elevated sTREM2 levels in CMD patients indicate its potential as a biomarker.

## 1. Introduction

Angina with no obstructive coronary artery disease (ANOCA) is increasingly recognized as a prevalent condition among individuals who experience angina-like symptoms [1]. A pivotal factor contributing to ANOCA is coronary microvascular dysfunction (CMD), which is characterized by the impaired structure or function of coronary microvessels [2,3]. CMD has garnered considerable attention due to its robust association with adverse cardiovascular events and elevated mortality rates [4,5]. The expert consensus of EAPC/ESC on INOCA in 2021 emphasizes that coronary microvascular dysfunction (CMD) as a unique clinical entity is a key pathophysiological mechanism of ANOCA, rather than a benign disease [6]. Furthermore, the 2024 ESC management guidelines propose an individualized treatment pathway for chronic coronary syndrome based on symptoms and microcirculatory functional phenotypes [7]. Therefore, the identification of CMD, along with subsequent targeted interventions, is associated with improvements in cardiovascular symptoms and overall prognosis.

**Figure 1:**
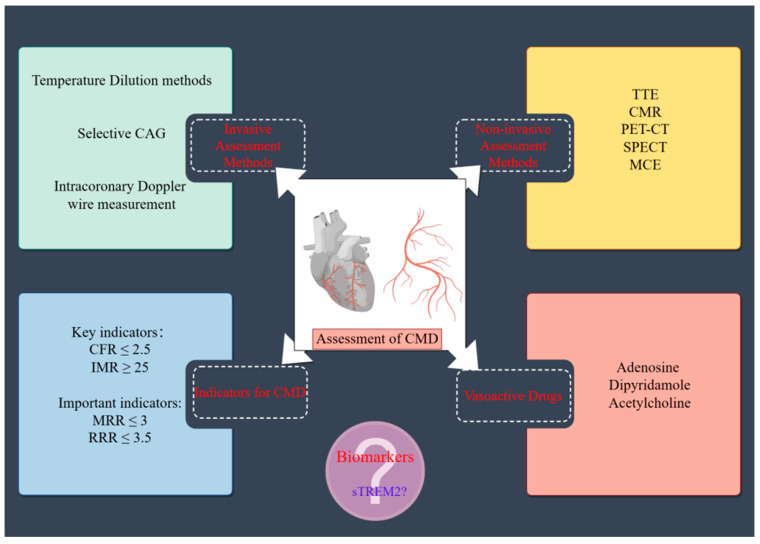
Conceptual diagram of CMD evaluation methods, along with relevant parameters and their diagnostic cutoff values. CMD, coronary microcirculation dysfunction; CAG, coronary angiography; TTE, transesophageal echocardiography; CMR, cardiac magnetic resonance; PET-CT, positron emission tomography–computed tomography; SPECT, single photon emission computed tomography; MCE, myocardial contrast echocardiography; CFR, coronary flow reserve; IMR, index of microvascular resistance; RRR, resistive reserve ratio; MRR, microvascular resistance reserve; sTREM2, soluble triggering receptor expressed on myeloid cells 2.

A diagnosis of CMD typically depends on invasive functional assessments of microcirculation [8], primarily encompassing coronary flow reserve (CFR) and the index of microvascular resistance (IMR). CFR is frequently employed to evaluate the overall coronary reserve, whereas the IMR predominantly reflects the functional status of the coronary microcirculation [9,10]. In addition to these established metrics, two emerging parameters—the resistive reserve ratio (RRR) and microvascular resistance reserve (MRR)—have been introduced to provide insights into coronary microcirculation. RRR functions as a comprehensive indicator of both flow and pressure levels assessed during resting and hyperemic states [11], while MRR remains unaffected by epicardial coronary disease and hemodynamic fluctuations [12,13] (Figure 1).

Nevertheless, these techniques are accompanied by certain limitations. Their invasive nature and operational complexities may pose risks to patients and may not be feasible in all clinical settings. Therefore, there is a pressing need for alternative, non-invasive diagnostic strategies to identify CMD in patients with ANOCA. The integration of biomarkers into clinical practice could enhance our ability to diagnose and manage CMD in individuals with ANOCA, addressing the shortcomings associated with current invasive testing methodologies.

Triggering Receptor Expressed on Myeloid Cells 2 (TREM2) is a transmembrane immune protein on myeloid cells, which is cleaved by metalloenzyme, producing soluble TREM2 (sTREM2) [14,15]. sTREM2 were initially thought to be associated with the progression of neurodegenerative disorders, such as Alzheimer’s disease [16,17]. Recent investigations have uncovered correlations between elevated sTREM2 levels and various cardiovascular diseases and outcomes (Table S1). Elevated sTREM2 levels have been identified as a potential biomarker for coronary heart disease [18]. Furthermore, sTREM2 has been recognized as a novel mediator of hypertensive heart failure through immune profiling of murine cardiac leukocytes [19]. Importantly, an observational study from the CATIS cohort has demonstrated that elevated levels of sTREM2 are independently correlated with increased risks of mortality and cardiovascular events following acute ischemic stroke [20]. Another cohort study has suggested that serum sTREM2 may serve as a predictor of cardiovascular death, potentially acting as a surrogate marker for plaque rupture [21]. However, the role of sTREM2 in CMD remains largely unexplored.

In this study, a comparative analysis was conducted on sTREM2 levels between patients with CMD and individuals without CMD, focusing on CFR, IMR, MRR, and RRR responses. Additionally, the correlation between sTREM2 and CMD was instigated to ascertain the potential of circulating sTREM2 levels as a predictive marker for CMD.

## 2. Methods

### 2.1. Study Population

**Figure 2:**
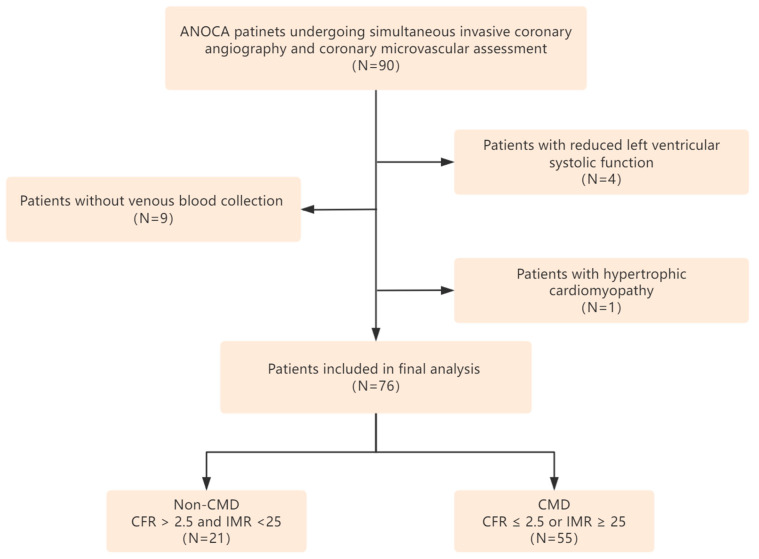
Study flowchart. ANOCA, non-obstructive coronary artery disease; CMD, coronary microcirculation dysfunction; CFR, coronary flow reserve; IMR, index of microvascular resistance.

This cross-sectional study included 76 patients diagnosed with ANOCA who underwent simultaneous invasive coronary angiography and coronary microvascular assessment at the China–Japan Friendship Hospital between July 2023 and May 2024. The inclusion criteria required participants to be over 18 years of age and to have unobstructed coronary arteries, defined as less than 50% diameter stenosis as assessed by visual inspection and a fractional flow reserve (FFR) greater than 0.80. Exclusion criteria included a recent myocardial infarction within the preceding three months, moderate to severe valvular heart disease, chronic kidney disease (with an estimated glomerular filtration rate of less than 45 mL/min/1.73 m^2^), primary cardiomyopathy, and reduced left ventricular systolic function (with an ejection fraction of less than 50%) (Figure 2).

The demographic characteristics recorded in this study include age, gender, body mass index (BMI), and smoking status. Additionally, laboratory parameters such as troponin T, N-terminal pro-B-type natriuretic peptide (NT-proBNP), alanine aminotransferase (ALT), fasting blood glucose (FBG), glycosylated hemoglobin (HbA1c), total cholesterol, triglycerides, low-density lipoprotein cholesterol (LDL-C), high-density lipoprotein cholesterol (HDL-C), creatinine, and estimated glomerular filtration rate (eGFR) were also documented. Furthermore, the medical history of participants was assessed, which included conditions such as diabetes, hypertension, hyperlipidemia, chronic kidney disease, chronic heart failure, and atrial fibrillation.

The study was conducted in the China–Japan Friendship Hospital (Registry Number: 2023-KY-077; Registry Date: 21 April 2023) and was registered at the international database ClinicalTrials.gov (Registry Name: Coronary Microvascular Dysfunction in Angina Patients With Non-obstructive Coronary Artery Disease (ANOCA-CMD); Registry Number: NCT06503640; Registry Date: 23 September 2022). It was conducted in compliance with the Declaration of Helsinki guidelines about ethical principles for medical research involving human subjects. Written informed consent was obtained by all patients before their participation in the study.

### 2.2. Evaluation of Microvascular Function

The thermodilution technique was employed to perform a thorough invasive evaluation of coronary function, as detailed in references [22,23]. To achieve maximal hyperemia, an intravenous infusion of adenosine was administered at a rate of 166 μg/kg/min. The mean transit time and the aortic and distal coronary pressures at rest and during hyperemia were recorded [24]. Various indices were derived from these measurements to assess microvascular function, including FFR, CFR, IMR, MRR, and RRR. FFR was calculated by determining the ratio of distal to proximal pressure, with an FFR value of ≥0.80 in the study vessel indicating the absence of flow limitation. IMR was defined as the product of hypermic mean transit time and distal coronary pressure; CFR was computed as the ratio of resting to hyperemic mean transit time [25]. MRR was defined as the product of CFR and the ratio of aortic pressure at rest to maxial hyperemia. RRR was calculated as the product of CFR and the ratio of mean distal coronary artery pressure at rest to that during hyperemia [26]. CMD was defined, based on non-invasive stress testing data and clinical outcomes from previous studies [27,28], as the presence of at least one abnormal parameter (CFR ≤ 2.5 or IMR ≥ 25). Additionally, MRR values of ≤3 or RRR values of ≤3.5 hold particular clinical significance in the evaluation of CMD [23,26].

### 2.3. Plasma Collection and sTREM2 Concentration Quantification

Venous blood samples were collected in the morning and subsequently centrifuged at 3000 rpm for a duration of 10 min. The plasma was then extracted and stored at −80 °C. The concentrations of sTREM2 were quantified utilizing human sTREM2 enzyme-linked immunosorbent (ELISA) kits (Laibio, Shanghai, China). All ELISA procedures were conducted using kits from the same batch and were performed in strict accordance with the manufacturer’s guidelines by trained technicians who were blinded to the clinical data.

### 2.4. Statistical Analysis

No other biological samples were collected except for the aforementioned venous blood collection. This study follows industry standards and uses the Research Electronic Data Capture (REDCap) database (https://114.116.255.168/redcap_v11.4.1/; accessed on 12 July 2024). An electronic case report form (eCRF) was built for clinical data collection and management, in order to accurately, timely, standardized, and complete disease data collection, ensure data structure and standardization, and patient personal information was removed to protect patient privacy. The scientific protocol was approved by the Ethics Committee of the China–Japan Friendship Hospital.

The normality of the data was assessed using the Kolmogorov–Smirnov test. Continuous variables that conformed to a normal distribution were reported as mean ± standard deviation. Differences between two groups were evaluated using the *t*-test, while one-way ANOVA was employed for comparisons involving multiple groups. Non-normally distributed variables were characterized by the median and interquartile ranges (IQR) and analyzed using the Mann–Whitney U test. Comparisons of count data were conducted using Chi-square tests and expressed as percentages. The Spearman correlation test was utilized to examine the association between sTREM2 levels and indices of microcirculation function. Logistic regression analysis was performed to evaluate the relationship between sTREM2 and CMD. The diagnostic accuracy of sTREM2 in identifying CMD patients was assessed using the receiver operating characteristic (ROC) curve. For analyses involving multiple comparisons, we applied the Bonferroni correction to control for the increased risk of Type I errors. All reported *p*-values in the subgroup analyses are the corrected *p*-values unless otherwise stated. A two-tailed *p*-value < 0.05 was considered statistically significant.

## 3. Results

### 3.1. Baseline Characteristics of the Study Population

In the present study, a total of 76 participants were enrolled, comprising 21 patients without CMD (non-CMD) and 55 patients with CMD. The baseline characteristics of the study cohort are detailed in Table 1. All participants demonstrated coronary arteries free of significant stenosis. The mean age of the CMD group was 64.8 years, with 54.5% being female, a distribution similar to that of the non-CMD group. There were no notable discrepancies inBMI, cardiac, hepatic, and renal function, lipid profiles, blood glucose levels, smoking status, or comorbidities between the two groups.

### 3.2. Plasma sTREM2 Level

**Figure 3:**
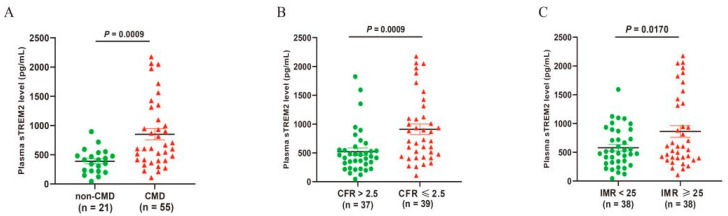
Plasma sTRME2 levels in participants with and without CMD (**A**), based on CFR Response (**B**), IMR Response (**C**). CMD, coronary microcirculation dysfunction; sTREM2, soluble triggering receptor expressed on myeloid cells 2; CFR, coronary flow reserve; IMR, index of microvascular resistance.

The plasma sTREM2 level in the CMD group exhibited a statistically significant increase compared to the non-CMD group (*p* = 0.0009, Figure 3A). Plasma sTREM2 concentration in patients based on CFR, IMR, MRR, or RRR alone were investigated further. We found that the plasma sTREM2 levels in patients with CFR ≤ 2.5 was significantly elevated compared to those with CFR > 2.5 (*p* = 0.0009, Figure 3B). Similarly, patients with IMR ≥ 25 had a significantly higher plasma sTREM2 level than those with IMR < 25 (*p* = 0.0170, Figure 3C). Moreover, individuals with MRR ≤ 3 exhibited a significantly higher plasma sTREM2 level than those with MRR > 3 (*p* = 0.0173, Figure S1A). Additionally, patients with RRR ≤ 3.5 demonstrated a significantly higher plasma sTREM2 level than those with RRR > 3.5 (*p* = 0.0004, Figure S1B).

### 3.3. Correlation Between sTREM2 and Indices for Coronary Microcirculation Function

**Figure 4:**
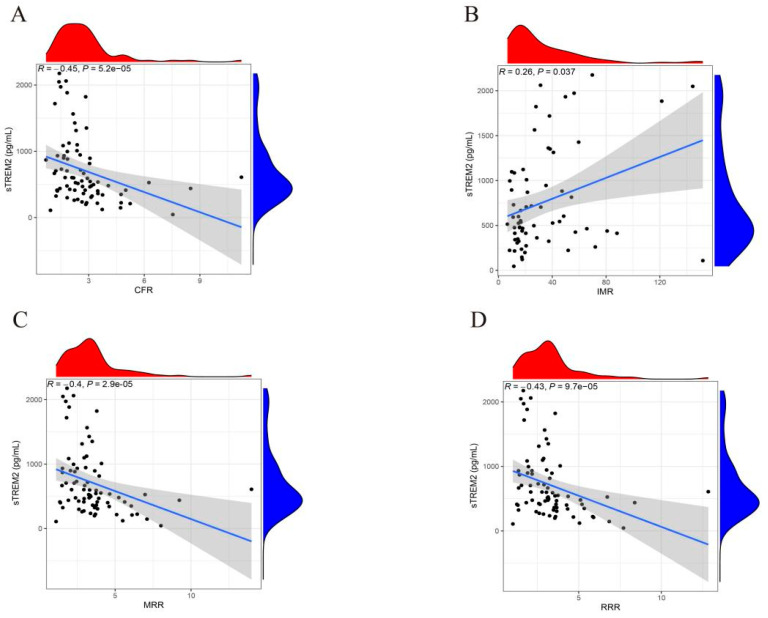
Correlation between plasma sTREM2 levels and CFR (**A**), IMR (**B**), MRR (**C**) and RRR (**D**). sTREM2, soluble triggering receptor expressed on myeloid cells 2; CFR, coronary flow reserve; IMR, index of microvascular resistance; MRR, microvascular resistance reserve; RRR, resistive reserve ratio.

Spearman correlation analysis was performed to investigate the liner correlation between sTREM2 and CMD. The results indicated that sTREM2 exhibited a positive correlation with CFR (*R* = −0.45, *p* =5.2 × 10^−5^, Figure 4A), MRR (*R* = −0.40, *p* = 2.9 × 10^−5^, Figure 4C) and RRR (*R* = −0.43, *p* = 9.7 × 10^−5^, Figure 4D), while demonstrating a positive correlation with IMR (*R* = 0.26, *p* = 0.037, Figure 4B).

### 3.4. Logistic Regression and Subgroup Analysis of Factors Influencing CMD

Univariable logistic regressions were performed on the variables presented in Table 2 to investigate their potential relationships with CMD. The association between sTREM2 and CMD was assessed using a multivariate logistic model, which was adjusted for confounding factors including age, gender, BMI, smoking, HbA1c, LDL-C, diabetes, hypertension, and hyperlipidemia. The findings revealed that elevated levels of sTREM2 were significantly and independently associated with an increased risk of CMD (OR = 1.003, 95% CI = 1.001–1.007, *p* = 0.008) (Table 3). Subgroup analyses were conducted to assess whether the diagnostic value of the sTREM2 remained consistent across diverse demographic characteristics or comorbidities. We found that the association between sTREM2 and CMD was significant and consistent in most subgroups except for in older adults (>60 years) or diabetic patients (Table S2).

### 3.5. The Diagnostic Value of sTREM2 for CMD

**Figure 5:**
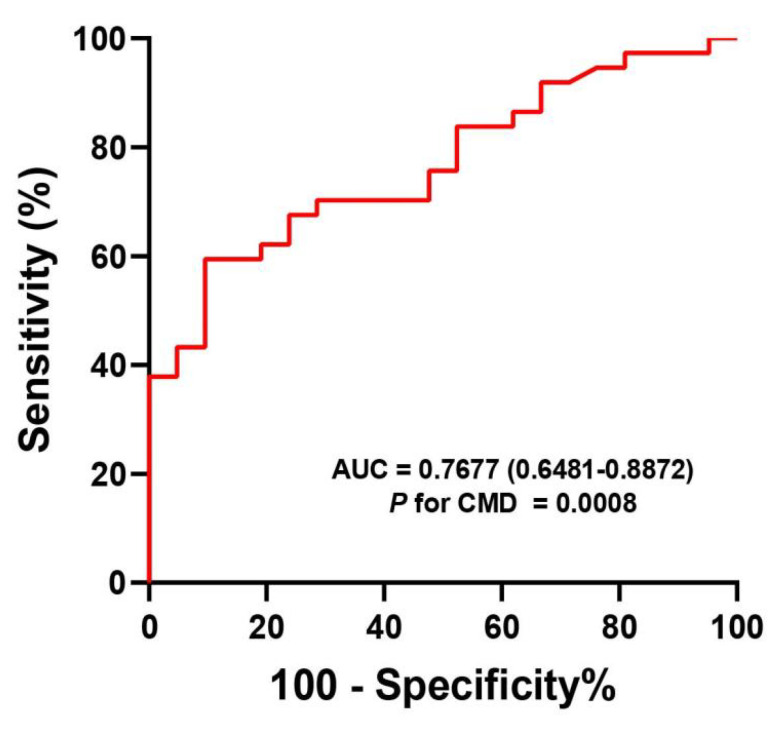
ROC analysis of sTREM2 in the diagnosis of CMD. CMD, coronary microcirculation dysfunction; sTREM2, soluble triggering receptor expressed on myeloid cells 2; ROC, receiver operating characteristic; AUC, area under the curve.

We conducted a comprehensive assessment of the diagnostic capability of sTREM2 in identifying CMD using ROC curves. The area under the curve (AUC) for plasma sTREM2 was calculated to be 0.7677 (95% CI: 0.6481–0.8872, *p* < 0.0001) (Figure 5). The established cut-off value for sTREM2 level was determined to be 595.5 pg/mL, with a sensitivity of 59.46% and a specificity of 90.48%. These findings suggest that sTREM2 has the potential to function as a biomarker for CMD.

### 3.6. Comparison of CMD Parameters Based on Grouping of sTRME2 Cut-Off Value

**Figure 6:**
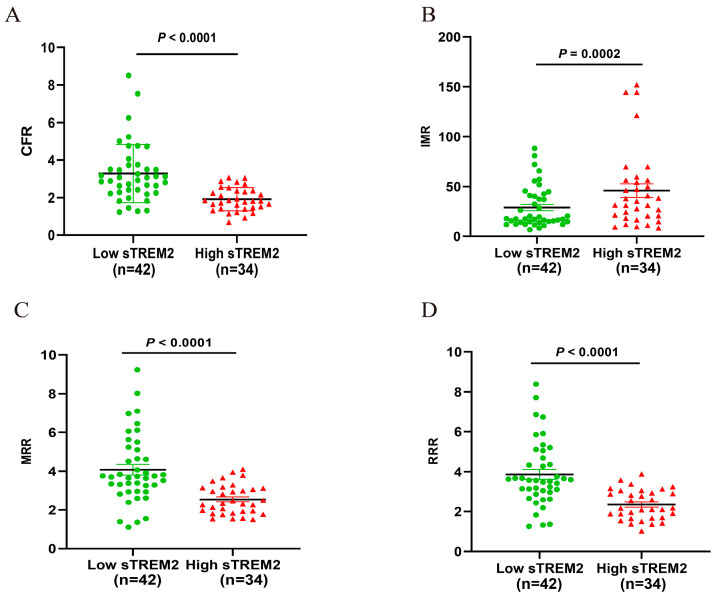
Comparison of microcirculation indices based on sTREM2 cut-off value, including CFR (**A**), IMR (**B**), MRR (**C**) and RRR (**D**). sTREM2, soluble triggering receptor expressed on myeloid cells 2; CFR, coronary flow reserve; IMR, index of microvascular resistance; MRR, microvascular resistance reserve; RRR, resistive reserve ratio.

We further classified the study population into two groups: a Low sTREM2 group (*n* = 42) and a High sTREM2 group (*n* = 34), based on the established cut-off value for sTREM2. Our analysis revealed that the CFR, MRR, and RRR of High sTREM2 group were significantly lower compared to the Low sTREM2 group, while the IMR was significantly higher in the High sTREM2 group (all *p* < 0.05, Figure 6).

## 4. Discussion

This cross-sectional study demonstrated that ANOCA patients with CMD had higher plasma levels of sTREM2 in comparison to those without CMD. Within the cohort of CMD patients, sTREM2 levels showed a positive correlation with IMR, and an inverse correlation with CFR, MRR, and RRR. Additionally, sTREM2 was identified as an independent predictor of CMD after controlling for confounding variables, and demonstrated substantial diagnostic accuracy. These findings provide novel insights into the association between sTREM2 and CMD, indicating the potential utility of sTREM2 as a biomarker for CMD.

The evaluation of coronary microvascular function utilizes both non-invasive and invasive techniques. Non-invasive imaging, including cardiac magnetic resonance imaging and positron emission tomography, offers safe, repeatable monitoring, but is limited by lower specificity, sensitivity, and reliance on qualitative data. In contrast, invasive physiological assessments provide direct, quantitative evaluation of microvascular function, enabling precise hemodynamic analysis. However, these methods carry risks such as bleeding, infection, and vascular injury, and are restricted to specific patient populations, limiting their broad application [29,30]. Nonetheless, these methodologies may not be appropriate for routine monitoring and screening purposes [31,32,33,34]. Serum biomarkers have the potential to offer supplementary prognostic information that extends beyond conventional risk factors and the Framingham Risk Score. However, there is no established serological marker for the diagnosis of CMD [35]. Therefore, the identification of such a marker is crucial for enhancing clinical practice.

Previous studies have shown that elevated sTREM2 levels are associated with atherosclerosis [36], coronary artery disease [18], and heart failure with preserved ejection fractions [19], suggesting its potential as a biomarker for cardiovascular disease severity and prognosis. Our study is the first to report significantly higher plasma sTREM2 levels in the CMD group compared to the non-CMD group. Notably, even when CMD is diagnosed based on a single parameter of coronary microcirculation function (CFR ≤ 2.5, IMR ≥ 25, MRR ≤ 3, or RRR ≤ 3.5), an elevation in sTREM2 levels is still observable. We conducted a linear correlation analysis between sTREM2 and observed a significant positive association with IMR, while showing a significant negative correlation with CFR, MRR, and RRR. These findings suggest a potential relationship between sTREM2 and the severity of CMD.

Although our research centers on sTREM2 in CMD, emerging evidence indicates that circulating sTREM2 could potentially act as a sensitive biomarker for chronic liver disease (CLD) across various stages, independent of surgical indications [37,38]. This underscores its broader role in detecting subclinical inflammation and tissue remodeling. In the context of CMD, elevated sTREM2 levels may similarly reflect early microvascular endothelial activation or macrophage-driven inflammation, potentially enabling earlier diagnosis before overt symptoms arise. However, unlike CLD, where sTREM2 correlates with liver fibrosis, its role in CMD likely involves distinct pathways linked to myocardial microvascular dysfunction. Future studies should explore whether sTREM2-guided interventions could improve outcomes in both hepatic and cardiovascular pathologies.

After controlling for potential confounding variables including age, sex, BMI, smoking status, LDL-C, HbA1c, eGFR and history of hypertension, diabetes and hyperlipidemia, sTREM2 levels were identified as an autonomous risk factor for CMD. Further subgroup analyses have revealed that the association between sTREM2 and CMD was significant and consistent in most subgroups except for in older adults or diabetic patients. The lack of significance in individuals > 60 years may reflect the influence of age-related comorbidities (e.g., advanced atherosclerosis, fibrosis) or competing inflammatory pathways that could obscure the specific role of sTREM2 in CMD. Additionally, older adults often exhibit more complex microvascular pathologies, which may dilute the biomarker signal. The attenuated association in diabetic individuals suggests that systemic metabolic disturbances (e.g., hyperglycemia, insulin resistance) may dominate the pathophysiology of microvascular dysfunction in this population, potentially overshadowing the contribution of sTREM2. Alternatively, diabetes-related endothelial dysfunction might involve distinct mechanisms that are less dependent on TREM2-mediated inflammation. Additionally, the diagnostic efficacy of sTREM2 for CMD was evaluated, revealing that it has good predictive accuracy for CMD. After grouping based on the cut-off value of sTREM2, it was found that ANOCA patients with high sTREM2 levels had more significant coronary microcirculation disorders. Consequently, sTREM2 could serve as a promising diagnostic marker for CMD, offering a novel perspective for clinical diagnosis and management.

The incorporation of sTREM2 levels as part of a non-invasive diagnostic panel could offer an important adjunct, allowing for earlier detection and monitoring of disease progression. Specifically, a multimodal approach integrating sTREM2 with established biomarkers, advanced imaging modalities, and invasive assessment could improve diagnostic accuracy, enhance risk stratification, and enable personalized management strategies in patients with suspected CMD [39] (Figure S2). Additionally, if validated, sTREM2 could guide therapies (e.g., anti-inflammatory agents or endothelial-modulating drugs) in CMD patients, enabling personalized treatment strategies. Furthermore, sTREM2 may serve as a surrogate endpoint in trials evaluating novel CMD therapies, accelerating drug development.

To the best of our knowledge, this study represents the first cross-sectional investigation carried out to establish the association between sTREM2 levels and CMD. We acknowledge the limitations inherent in this study. Firstly, the single-center design may limit the generalizability of the findings, highlighting the need for validation in multicenter cohorts. Secondly, the cross-sectional design of the study makes it challenging to determine the precise causal relationship between plasma sTREM2 levels and CMD. Larger prospective studies with more extensive sample sizes are necessary to validate this relationship. Thirdly, despite adjusting for various confounding factors such as age and gender, it is possible that the research findings could still be influenced by other potential confounders. Fourthly, the exclusion of patients with left ventricular systolic dysfunction may restrict the applicability of our findings to broader heart failure populations. Future research should aim to address this gap by including patients across a wider spectrum of left ventricular function. Fifthly, we did not assess dynamic changes in sTREM2 over time. Future works should incorporate serial sTREM2 measurements to determine whether fluctuations predict disease progression or treatment response.

In conclusion, our study revealed a significant increase in sTREM2 levels among patients with CMD. Moreover, elevated sTREM2 levels were found to be positively correlated with the severity of CMD. Furthermore, sTREM2 levels emerged as an independent risk factor for CMD and exhibited a good diagnostic capability in detecting CMD.

## Figures and Tables

**Table 1:** Baseline characteristics for ANOCA patients with and without CMD.

Variables	Non-CMD (*n* = 21)	CMD (*n* = 55)	*p*-Value
Age, y	62.9 ± 10.8	64.8 ± 11.9	0.533
Female Gender, *n* (%)	11 (52.4)	30 (54.5)	0.866
BMI, kg/m^2^	25.87 ± 3.63	25.61 ± 3.18	0.770
Current smoking, *n* (%)	3 (14.3)	14 (25.5)	0.296
Diabetes, *n* (%)	5 (23.8)	19 (34.5)	0.368
Hypertension, *n* (%)	13 (61.9)	35 (63.6)	0.889
Hyperlipidemia, *n* (%)	19 (90.5)	41 (75.9)	0.157
Chronic kidney disease, *n* (%)	1 (4.8)	1 (1.8)	0.473
Chronic heart failure, *n* (%)	1 (4.8)	1 (1.8)	0.534
Atrial fibrillation, *n* (%)	0 (0)	2 (3.6)	0.822
Troponin T, ng/mL	0.01 ± 0.004	0.01 ± 0.012	0.261
NT-proBNP, pg/mL	201.15 (32.0, 170.7)	133.23 (31.7, 131.0)	0.369
ALT, IU/L	29.75 (15.0, 37.0)	24.35 (16.0, 29.0)	0.225
Fasting blood glucose, mmol/L	6.15 ± 1.68	6.94 ± 2.24	0.149
HbA1c, %	6.08 ± 0.81	6.40 ± 1.04	0.228
Total cholesterol, mmol/L	4.17 ± 0.92	3.95 ± 0.93	0.354
Triglyceride, mmol/L	1.37 ± 0.71	1.47 ± 0.72	0.559
LDL-C, mmol/L	2.52 ± 0.72	2.36 ± 0.72	0.401
HDL-C, mmol/L	1.32 ± 0.27	1.26 ± 0.26	0.392
Creatinine, μmol/L	66.56 (57.3, 76.8)	74.11 (50.9, 78.3)	0.659
eGFR, mL/min/1.73 m^2^	91.9 (85.7, 104.0)	93.4 (85.4, 101.3)	0.722
FFR	0.89 ± 0.03	0.90 ± 0.04	0.084

ANOCA, angina with no obstructive coronary artery disease; CMD, coronary microvascular dysfunction; BMI, body mass index; NTpr-BNP, N-terminal pro-B-type natriuretic peptide; ALT, alanine aminotransferase; FBG, fasting blood glucose; HbA1c, hemoglobin A1c; LDL-C, low-density lipoprotein cholesterol; HDL-C, high-density lipoprotein cholesterol; eGFR, estimates of glomerular filtration rate; FFR, fractional flow reserve.

**Table 2:** Univariable logistic regression association between sTREM2 and CMD.

Variable	CMD	
	OR (95%CI)	*p*-Value
Age	1.014 (0.971–1.059)	0.527
Gender	1.091(0.398–2.988)	0.866
BMI	0.977 (0.839–1.138)	0.767
Smoking	1.319 (0.328–5.298)	0.696
LDL-C	0.744 (0.375–1.476)	0.397
HbA1c	1.464 (0.786–2.729)	0.230
eGFR	1.006 (0.975–1.037)	0.718
sTREM2	1.004 (1.001–1.006)	0.003

CMD, coronary microvascular dysfunction; BMI, body mass index; LDL-C, low density lipoprotein cholesterol; sTREM2, soluble triggering receptor expressed on myeloid cells 2; HbA1c, glycosylated hemoglobin; eGFR, estimated glomerular filtration rate; OR, odds ratio; CI, confidence interval.

**Table 3:** Multivariable logistic regression association between sTREM2 and CMD.

Variable	CMD	
	OR (95% CI)	*p*-Value
sTREM2	1.003 (1.001–1.007)	0.008
Age	0.985 (0.871–1.114)	0.527
Gender	4.324 (0.501–37.312)	0.183
BMI	1.023 (0.770–1.047)	0.770
Smoking	6.163 (0.590–64.374)	0.129
LDL-C	0.436 (0.148–1.284)	0.132
HbA1c	2.553 (0.418–15.612)	0.310
eGFR	0.990 (0.913–1.073)	0.803
Diabetes	0.301 (0.013–7.204)	0.459
Hypertension	1.163 (0.163–8.296)	0.670
Hyperlipidemia	0.223 (0.014–3.646)	0.293

Definitions and abbreviations are consistent with those in Table 2.

## Data Availability

The original contributions presented in this study are included in the article/Supplementary Materials. Further inquiries can be directed to the corresponding authors.

## Supplementary Materials

The following supporting information can be downloaded at: https://www.mdpi.com/article/10.3390/jcm14061816/s1, Figure S1: Plasma sTRME2 levels in participants based on MRR Response (A), RRR Response (B). sTREM2, soluble triggering receptor expressed on myeloid cells 2; MRR, microvascular resistance reserve; RRR, resistive reserve ratio; Figure S2: The potential role of integrating sTREM2 testing, advanced cardiovascular imaging, and invasive functional assessments in the diagnosis and management of CMD patients. CMD, coronary microcirculation dysfunction; sTREM2, soluble triggering receptor expressed on myeloid cells 2; Table S1: Studies on Elevated sTREM2 Levels in Cardiovascular Diseases; Table S2: Association Between sTREM2 and CMD Stratified by Demographic and Clinical Subgroups.
