# Supplementary material for: Single-Center Analysis of Soluble TREM2 as a Biomarker in Coronary Microvascular Dysfunction: A Cross-Sectional Study
Source: J Clin Med. 2025 Mar 7;14(6):1816. doi: 10.3390/jcm14061816 (PMC11942759; doi:10.3390/jcm14061816)
Supplement: Supplementary file 1 [file jcm-14-01816-s001.zip › jcm-3426039-supplementary.pdf]

Table S1. Studies on Elevated sTREM2 Levels in Cardiovascular Diseases

**Study (year)**	**Study Design**	**Population**	**Key Findings**	**Related Disease**
Liu W et al. (2023)	Cross-sectional study	86 patients with CAD	Higher sTREM2 levels associated with CAD occurrence	CAD
Cuciuc, Valeri et al. (2022)	Retrospective cohort	230 patients with CAD	Elevated sTREM2 correlated with cardiovascular death	CAD
Lu, Yaling et al. (2022)	Retrospective cohort	3285 patients with AIS	Increased sTREM2 lined to 1-year death, cardiovascular events and severe disability	AIS
Moore, Elizabeth E et al. (2021)	Cross-sectional study	146 elderly people	Higher sTREM2 associated with aortic stiffness	aortic stiffness
Smart, Charles Duncan et al. (2023)	Cross-sectional and mechanistic studies	9 patients with HFpEF	Higher sTREM2 associated with HFpEF occurrence	HFpEF

CAD, coronary artery disease; AIS, acute ischemic stroke; HFpEF, heart failure with preserved ejection fraction; sTREM2, soluble triggering receptor expressed on myeloid cells 2.

Table S2. Association Between sTREM2 and CMD Stratified by Demographic and Clinical Subgroups

Variables	n (%)	OR (95%CI)	*P*	P for interaction
Sex				0.990
Male	35 (46.05)	1.011 (1.009 ~ 1.041)	**0.044**	
Female	41 (53.95)	1.00 5(1.001 ~ 1.012)	**0.028**	
Age				0.777
< 60 years	23 (30.26)	1.006 (0.999 ~ 1.017)	0.096	
≥ 60 years	53 (69.74)	1.005 (1.002 ~ 1.016)	**0.017**	
BMI				0.226
< 24 kg/m^2	26 (34.21)	1.012 (1.001 ~ 1.019)	**0.046**	
≥ 24 kg/m^2	50 (65.79)	1.00 5(1.002 ~ 1.018)	**0.023**	
Diabetes				0.206
NO	52 (68.42)	1.01 (1.00 ~ 1.01)	**0.006**	
YES	24 (31.58)	1.00 (0.99 ~ 1.01)	0.170	
hypertension				0.187
NO	28 (36.84)	1.01 (1.00 ~ 1.01)	**0.034**	
YES	48 (63.16)	1.00 (1.00 ~ 1.01)	**0.037**	

BMI, body mass index; CMD, coronary microcirculation dysfunction; sTREM2, soluble triggering receptor expressed on myeloid cells 2; OR, odds ratio; CI, confidence interval.

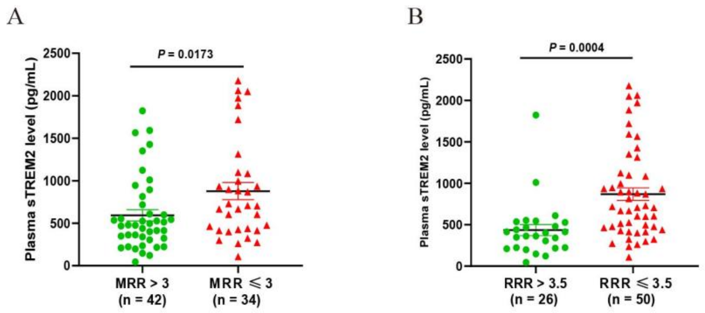

Figure S1. Plasma sTRME2 levels in participants based on MRR Response (A), RRR Response (B). sTREM2, soluble triggering receptor expressed on myeloid cells 2; MRR, microvascular resistance reserve; RRR, resistive reserve ratio.

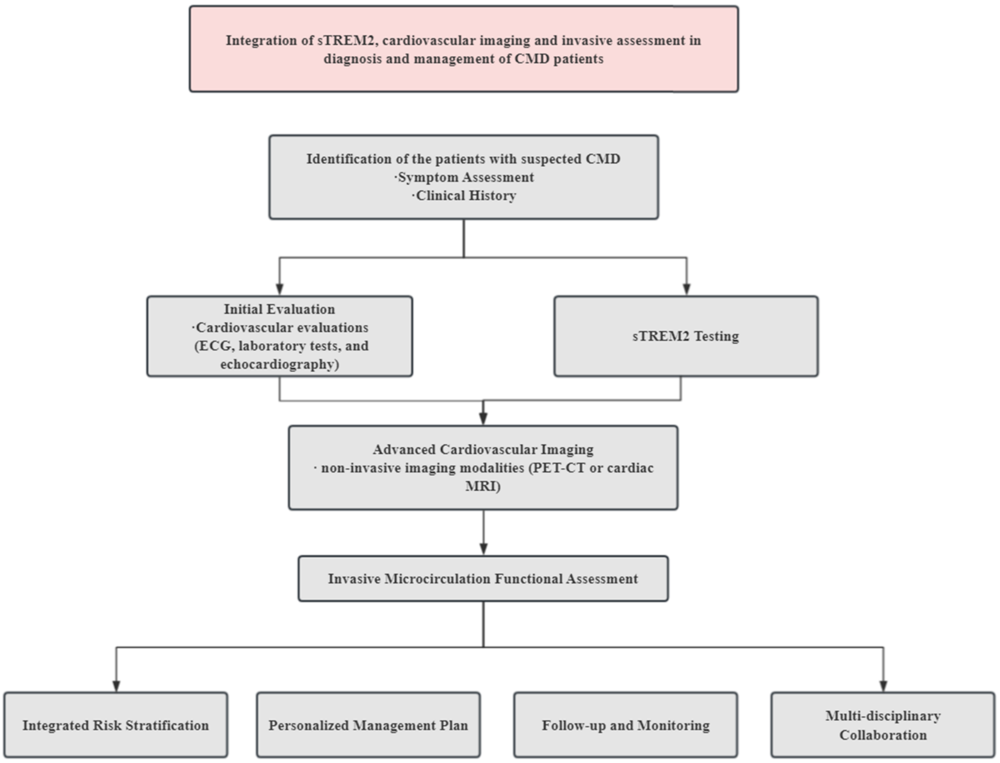

Figure S2. The potential role of integrating sTREM2 testing, advanced cardiovascular imaging, and invasive functional assessments in the diagnosis and management of CMD patients. CMD, coronary microcirculation dysfunction; sTREM2, soluble triggering receptor expressed on myeloid cells 2; ECG; Electrocardiogram; PET-CT, positron emission tomography-computed tomography; MRI, magnetic resonance imaging.
